# Supplementary material for: JMJD3 and UTX determine fidelity and lineage specification of human neural progenitor cells
Source: Nat Commun. 2020 Jan 20;11:382. doi: 10.1038/s41467-019-14028-x (PMC6971254; doi:10.1038/s41467-019-14028-x)
Supplement: Supplementary file 1 — Supplementary Information [file 41467_2019_14028_MOESM1_ESM.pdf]

JMJD3 and UTX Determine Fidelity and Lineage Specification of Human Neural Progenitor Cells

Shan et al.

Supplementary Figure 1

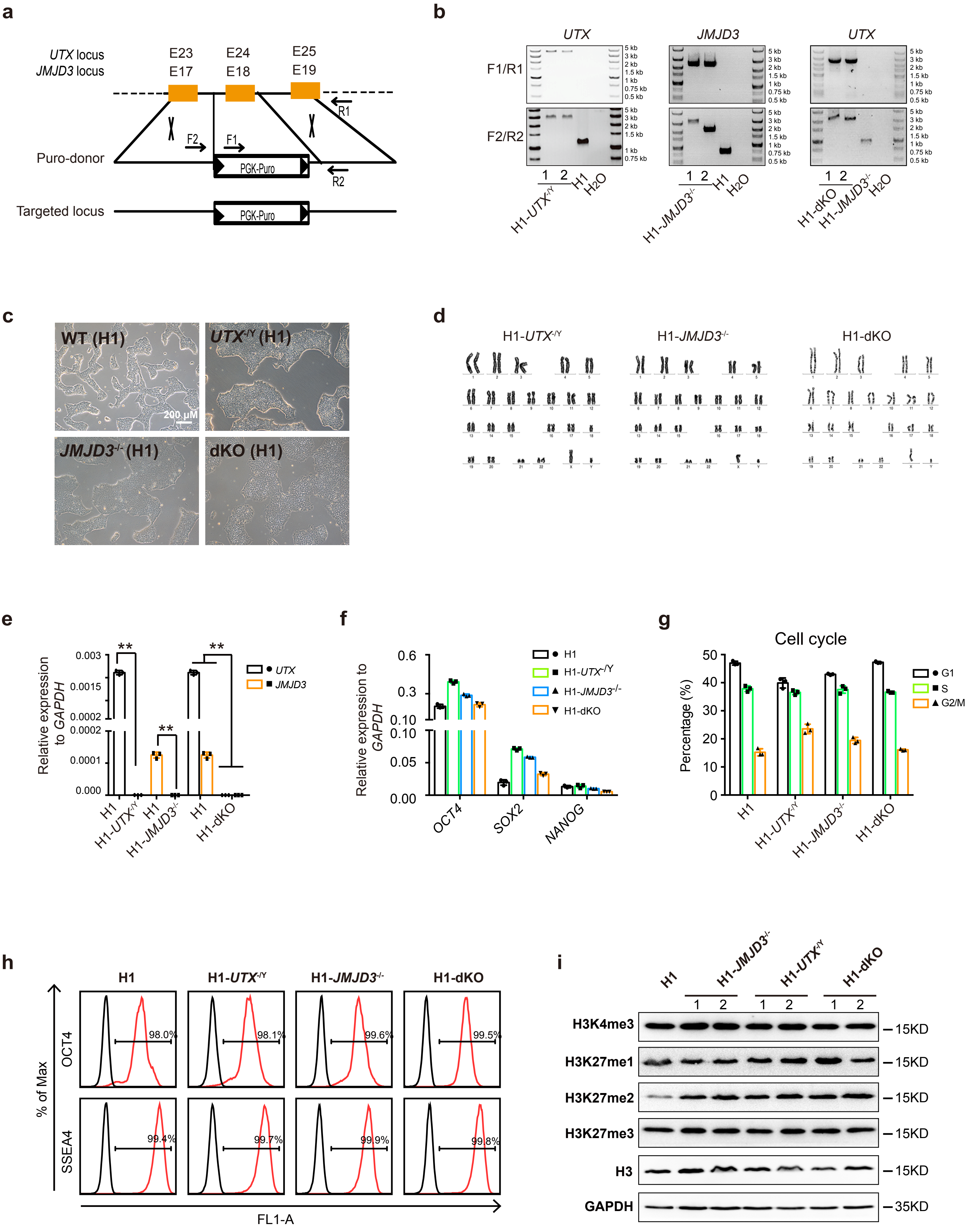

Supplementary Figure 1 Deletion of KDM6s in human embryonic stem cells
a. Overview of the gene targeting strategy for *UTX* and *JMJD3*. Key catalytic sites of JmjC domain of UTX and JMJD3 were deleted. Key catalytic sites of JmjC domain are in exon 24 (E24) of UTX and exon 18 (E18) of JMJD3, respectively. **b.** PCR identification of H1 hESCs with targeted deletion of *UTX*, *JMJD3*, and *JMJD3*/*UTX*. Wild type (WT) H1 hESCs serve as a negative control. hESCs, human embryonic stem cells. **c.** Morphology of H1 hESCs with targeted deletion of *UTX*, *JMJD3*, and *JMJD3*/*UTX* (dKO). Scale bar, 200 μm. **d.** Karyotype of H1 hESCs with targeted deletion of *UTX*, *JMJD3*, and *JMJD3*/*UTX* (dKO). **e.** qRT-PCR analysis on the expression level of targeted genes *UTX* and *JMJD3* in KDM6-deficient hESCs (H1-$UTX^{-/Y}$, H1-$JMJD3^{-/-}$, H1-dKO). Wild type H1 hESCs serve as control. Significance level were determined using unpaired two-tailed Student's t-tests. **, P <0.01. The data represent mean ± SD (standard deviation) from three independent repeats (n=3). **f.** qRT-PCR analysis on the expression level of pluripotent genes *OCT4*, *SOX2*, and *NANOG* in KDM6-deficient hESCs (H1-$UTX^{-/Y}$, H1-$JMJD3^{-/-}$, H1-dKO). Wild type H1 hESCs serve as control. The data represent mean ± SD from three independent repeats (n=3). **g.** Cell cycle assay for three KDM6 mutant hESCs, respectively. Wild type H1 serve as control. The data represent mean ± SD from three independent repeats (n=3). **h.** FACS analysis on the expression of indicated pluripotent markers OCT4 and SSEA4 in the indicated hESCs. **h.** Total level of the indicated histone modifications in gene targeted cells. The total histone modification level was analyzed by western-blot using the specific antibody on the whole cell lysates from each indicated cell line. All error bars throughout the figure represent SD (standard deviation) from three independent repeats (n=3). Source data are provided as a Source Data file. Related to Figure 1.

Supplementary Figure 2

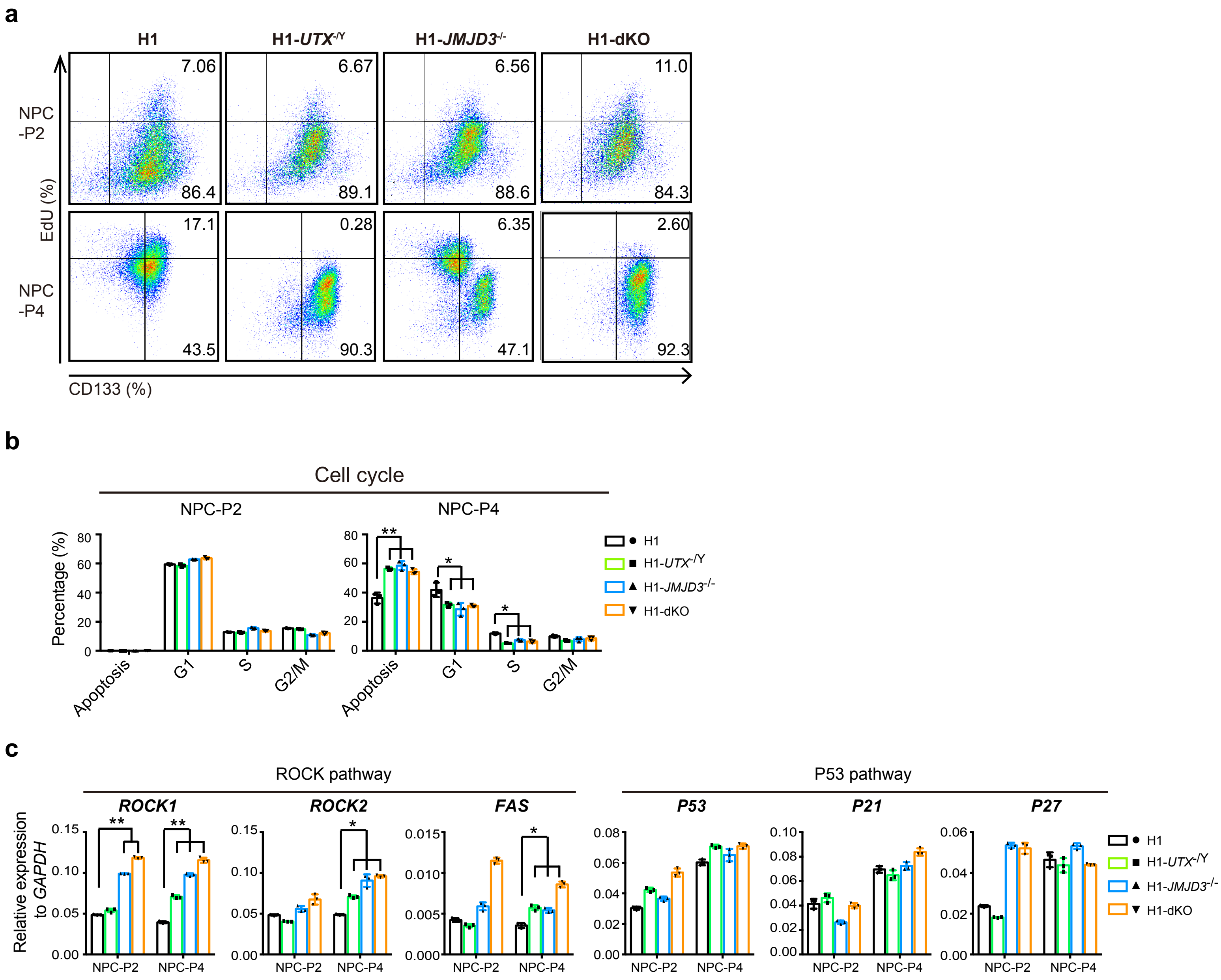

Supplementary Figure 2 KDM6s maintain the proliferation ability of human NPCs
a. FACS result of EdU insertion assay for $CD133^+$ three KDM6 mutant NPCs lacking UTX or JMJD3 or both at passage 2 (P2) and passage 4 (P4), respectively. dKO, deletion of both *JMJD3* and *UTX*. NPC, neural progenitor cell. **b.** Cell cycle assay for three KDM6 mutant NPCs at passage 2 (P2) and passage 4 (P4), respectively. Wild type H1 serve as control. Significance level was determined using unpaired two-tailed Student's t-tests. **, *P* <0.01. The data represent mean ± SD (standard deviation) from three independent repeats (n=3). **c.** qRT-PCR analysis on the expression level of ROCK pathway *ROCK*1, *ROCK2*, *FAS* and P53 pathway genes *P53*, *P21*, *P27* in three KDM6 mutant NPCs at passage 2 (P2) and passage 4 (P4), respectively. Wild type H1 serve as control. Significance level was determined using unpaired two-tailed Student's t-tests. *, *P* <0.05. **, *P* <0.01. The data represent mean ± SD from three independent repeats (n=3). All error bars throughout the figure represent SD (standard deviation) from three independent repeats (n=3). Source data are provided as a Source Data file. Related to Figure 2.

Supplementary Figure 3

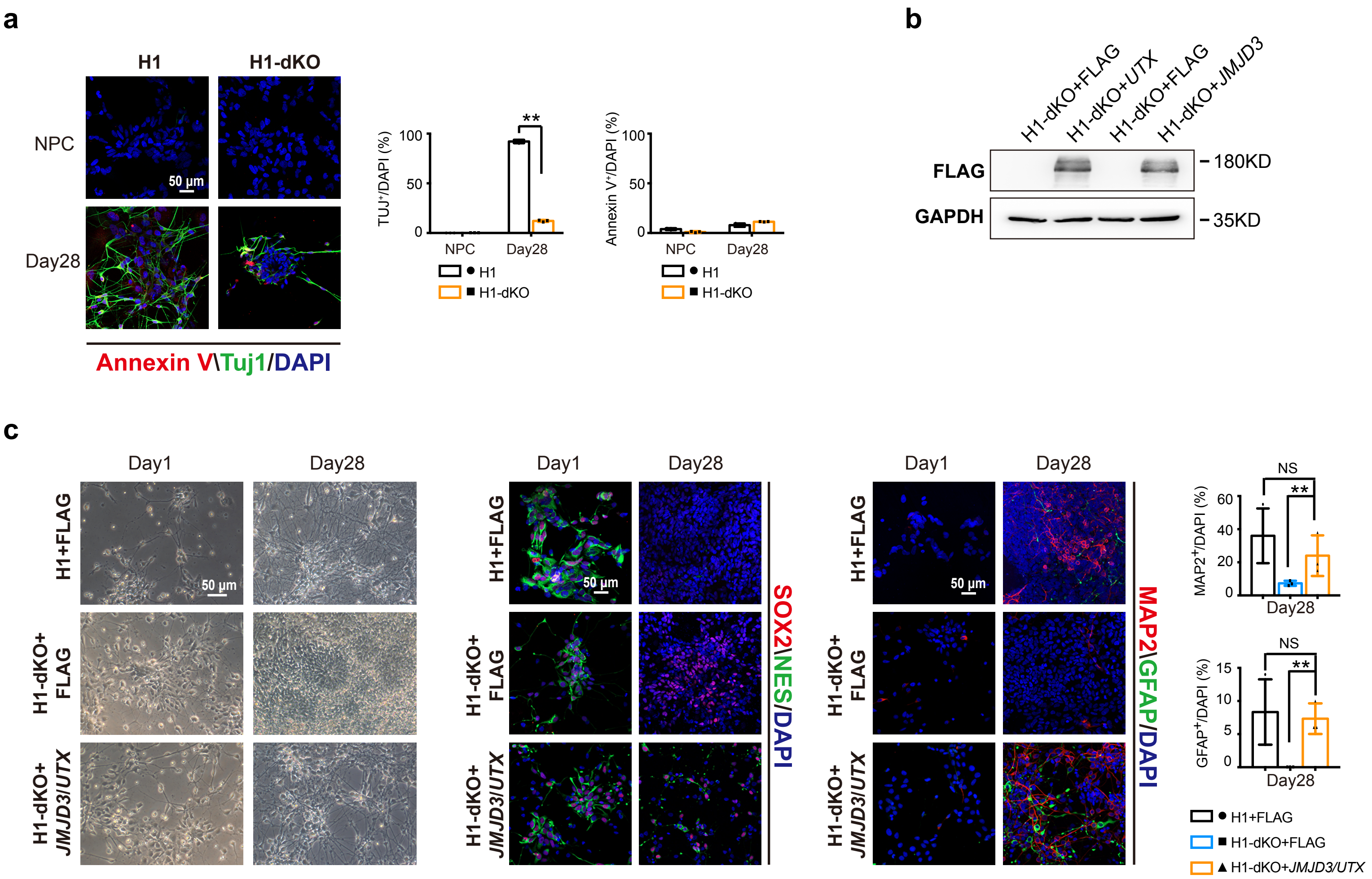

Supplementary Figure 3 KDM6 over-expression rescues the neuron/glia defect **a.** Immunostaining on the neuronal marker TUJ1, apoptosis marker Annexin V in the indicted undifferentiated or differentiated NPCs. Scale bar, 50 μm. Quantity data of TUJ^+ or Annexin V^+ cells were analyzed. Significance level was determined by unpaired two-tailed Student's t-tests. **, *P* <0.01. The data represent mean ± SD (standard deviation) from three independent replicates (n=3). WT, wild type. dKO, deletion of both *JMJD3* and *UTX*. **b.** Western-blot for UTX-FLAG or JMJD3-FLAG with FLAG antibody in dKO hES cells with UTX-FLAG-OE or JMJD3-FLAG-OE, respectively. dKO, deletion of both JMJD3 and UTX. **c.** Morphology, NPC markers SOX2/NES immuno-staining, neuron and glia markers MAP2/GFAP immune-staining assay in forced expression of *JMJD3/UTX* in dKO-NPCs (H1-dKO+*JMJD3/UTX*) during random differentiation at day1 (Day1) and day 28 (Day28), respectively. Quantity data of $MAP2^+$ or $GFAP^+$ cells were analyzed. Significance level was determined by unpaired two-tailed Student's t-tests. **, *P* <0.01. NS, no significance. The data represent mean ± SD from three independent replicates (n=3). Overexpression of FLAG in H1 (H1+FLAG) and dKO (H1-dKO+FLAG) cells serve as positive control and negative control, respectively. Scale bar, 50 μm. All error bars throughout the figure represent SD (standard deviation) from three independent repeats (n=3). Related to Figure 3.

Supplementary Figure 4

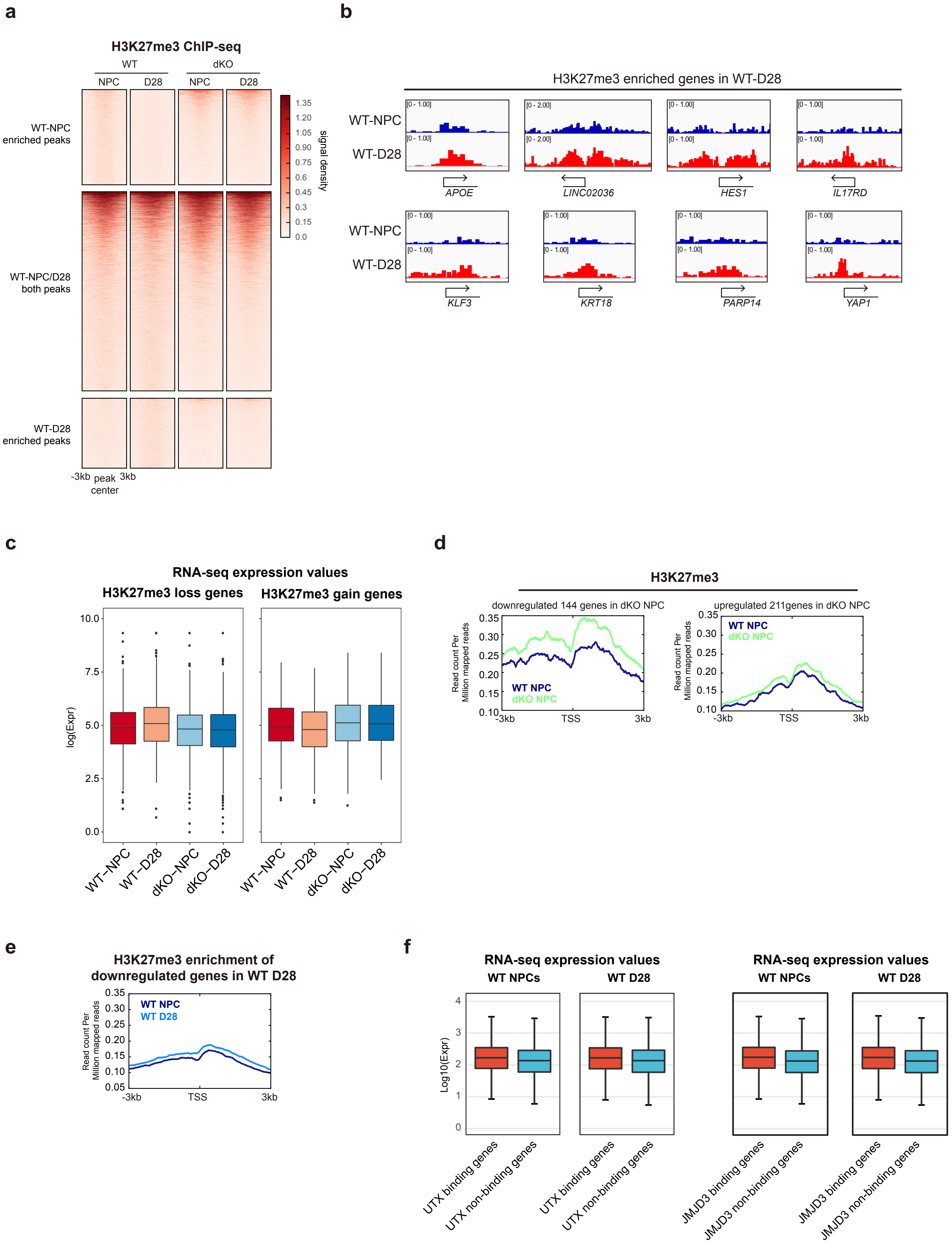

Supplementary Figure 4 KDM6s deficiency leads to H3K27me3 accumulation
a. Heatmap of H3K27me3 ChIP-seq for WT-NPC enriched peaks compared with WT-D28, WT-NPC/D28 both shared peaks, WT-D28 H3K27me3 enriched peaks compared with WT-NPC from NPC to D28 in WT and dKO cells, respectively. WT, wild type. dKO, deletion of both JMJD3 and UTX. NPC, neural progenitor cell. **b.** Genomic views of selected H3K27me3 enriched genes in wild-type D28 differentiated cells. **c.** Box-plot of expression value for the indicated genes described in the indicated cells. **d.** H3K27me3 enrichment analysis for downregulated or upregulated genes in dKO NPC cells compared with WT NPC cells. **e.** H3K27me3 enrichment analysis for downregulated genes in wild-type D28 differentiated cells compared with WT NPC cells. **f.** Box-plot of expression value for UTX or JMJD3 binding or non-binding sites in the indicated cells, respectively. Related to Figure 6.

Supplementary Figure 5

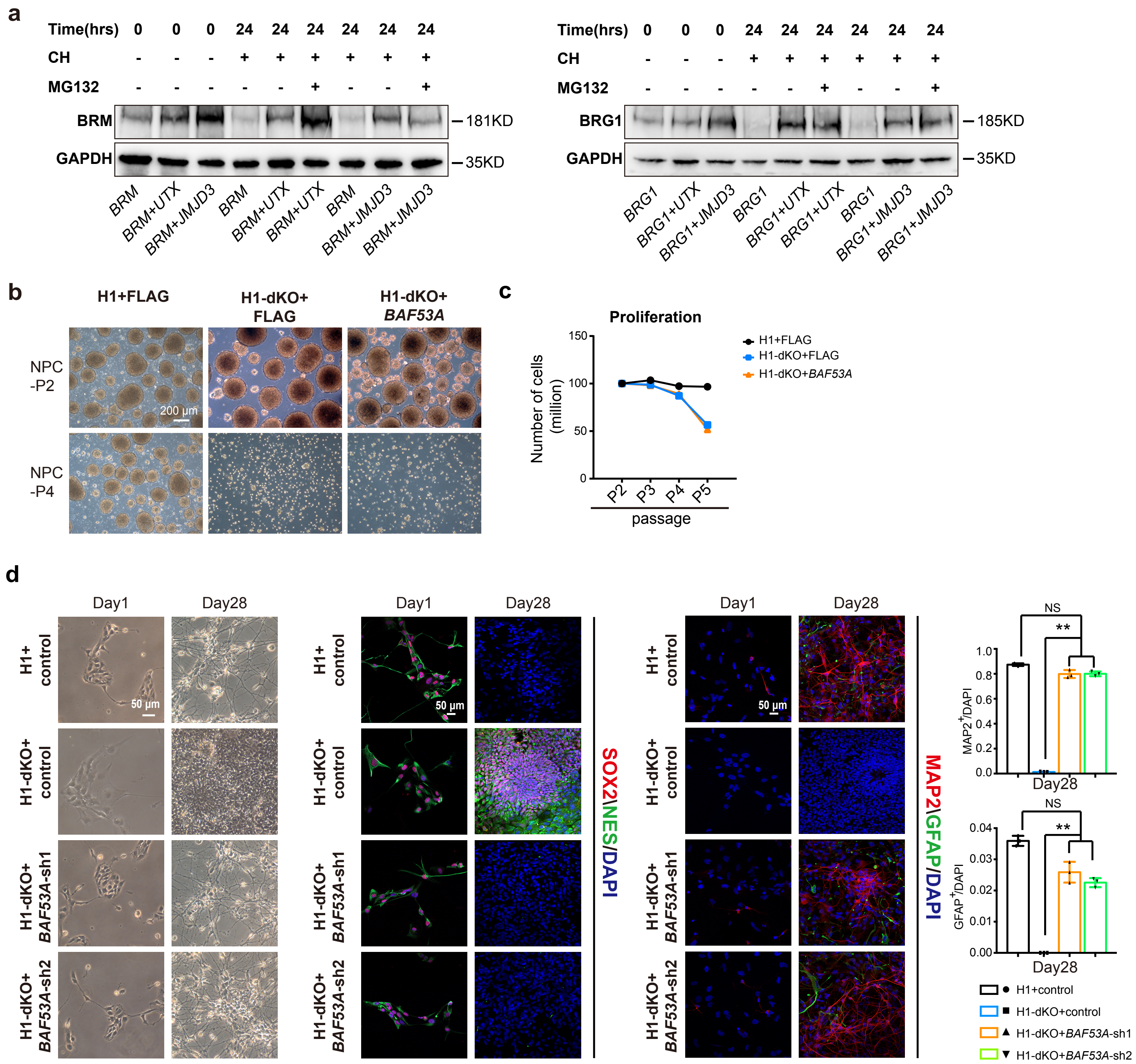

Supplementary Figure 5 BAF53B rescues neuron/glia defect in KDM6-dKO NPCs
a. Left panel, western blot analysis of BRM in 293T plus Cycloheximide (CH) or/and MG132 with overexpression of BRM, BRM and UTX, or BRM and JMJD3, respectively. Right panel, western blot analysis of BRG1 in 293T plus Cycloheximide (CH) or/and MG132 with overexpression of BRG1, BRG1 and UTX, or BRG1 and JMJD3, respectively. Cycloheximide (CH), a protein synthesis inhibitor. MG132, a proteasome inhibitor. **b.** Morphology of indicated NPCs maintained as neural spheres at passage 2 (P2) or passage 4 (P4). Scale bar, 200 μm. dKO, deletion of both JMJD3 and UTX. NPC, neural progenitor cell. **c.** Proliferation curve of the indicated NPCs at different passages. The data represent mean ± SD (standard deviation) from three independent replicates (n=3). **d.** Morphology, NPC markers SOX2/NES immuno-staining, neuron and glia markers MAP2/GFAP immune-staining assay in knock-down expression of *BAF53A* in dKO-NPCs by shRNA (H1-dKO+*BAF53A*-sh1 and H1-dKO+*BAF53A*-sh2) during random differentiation at day1 (Day1) and day 28 (Day28), respectively. Quantity data of $MAP2^+$ or $GFAP^+$ cells were analyzed. Significance level was determined by unpaired two-tailed Student's t-tests. **, P <0.01. NS, no significance. The data represent mean ± SD from three independent replicates (n=3). WT (H1+control) and dKO (H1-dKO+control) cells serve as positive control and negative control, respectively. WT, wild type. Scale bar, 50 μm. All error bars throughout the figure represent SD (standard deviation) from three independent repeats (n=3). Related to Figure 7.

Supplementary Figure 6

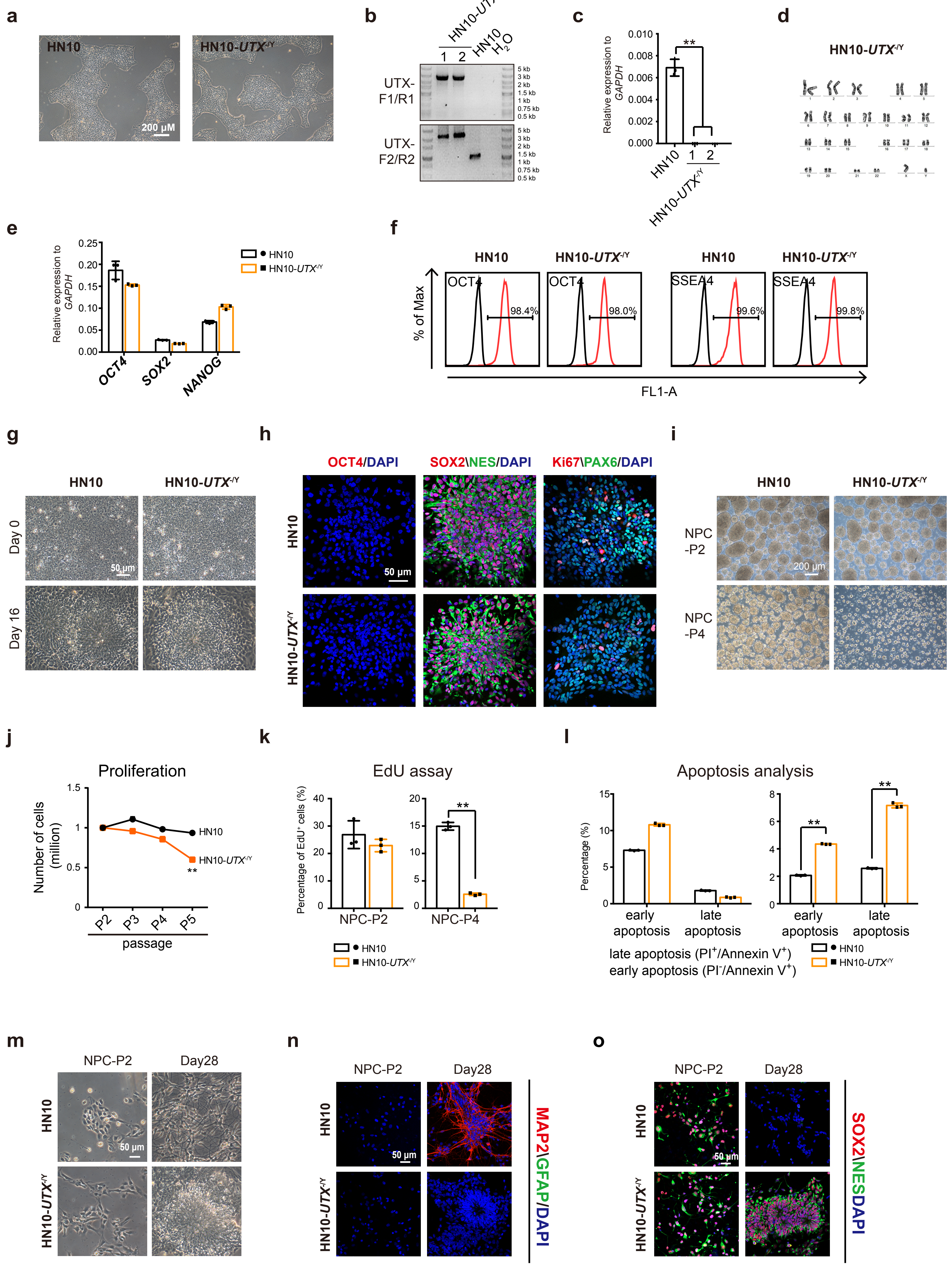

Supplementary Figure 6 None of *UTX* in HN10 cells fails to generate neuron/glia **a.** Morphology of HN10 hESCs with targeted deletion of *UTX*. Scale bar, 200 μm. **b.** PCR identification of HN10-$UTX^{-/Y}$ hESCs. **c.** qRT-PCR analysis for *UTX* in HN10-$UTX^{-/Y}$. Significance level were determined using unpaired two-tailed Student's t-tests. **, *P* <0.01. The data represent mean ± SD (standard deviation) from three independent repeats (n=3). **d.** Karyotype of HN10-$UTX^{-/Y}$. **e.** qRT-PCR analysis for *OCT4*, *SOX2*, and *NANOG* in HN10-$UTX^{-/Y}$ hESCs. The data represent mean ± SD from three independent repeats (n=3). **f.** FACS analysis for OCT4 and SSEA4 in the indicated hESCs. **g.** Morphology of neural differentiation from HN10-$UTX^{-/Y}$ hESCs at Day 0 and Day16, respectively. Scale bar, 50 μm. **h.** Immunostaining on the pluripotent marker OCT4, NPC markers SOX2/NES/PAX6, and proliferation marker Ki67 in wild type and *UTX*-deficient HN10-NPCs. Scale bar, 50 μm. **i.** Morphology of *UTX*-deficient HN10-NPCs at passage 2 (P2) and passage 4 (P4), respectively. Scale bar, 200 μm. **j.** Proliferation curve of *UTX*-deficient HN10-NPCs. Significance level was determined using unpaired two-tailed Student's t-tests. **, *P* <0.01. The data represent mean ± SD from three independent repeats (n=3). **k.** EdU insertion assay for *UTX*-deficient HN10-NPCs at passage 2 (P2) and passage 4 (P4), respectively. Significance level was determined using unpaired two-tailed Student's t-tests. **, *P* <0.01. The data represent mean ± SD from three independent repeats (n=3). **l.** Percentage of Early and late apoptosis in *UTX*-deficient HN10-NPCs at passage 2 (P2) and passage4 (P4), respectively. Significance level was determined using unpaired two-tailed Student's t-tests. **, *P* <0.01. The data represent mean ± SD from three independent repeats (n=3). **m.** Morphology of *UTX*-deficient HN10-NPCs (NPC-P2) and their differentiated cells at day 28 (Day28), respectively. Scale bar, 50 μm. **n.** Immunostaining on the neuronal marker MAP2, glia marker GFAP in *UTX*-deficient HN10-NPCs and Day28. Scale bar, 50 μm. **o.** Immunostaining on the NPC markers SOX2/NES in *UTX*-deficient HN10-NPCs and Day28. Scale bar, 50 μm. All error bars throughout the figure represent SD (standard deviation) from three independent repeats (n=3). Source data are provided as a Source Data file.

Supplementary Figure 7

Figure 1e

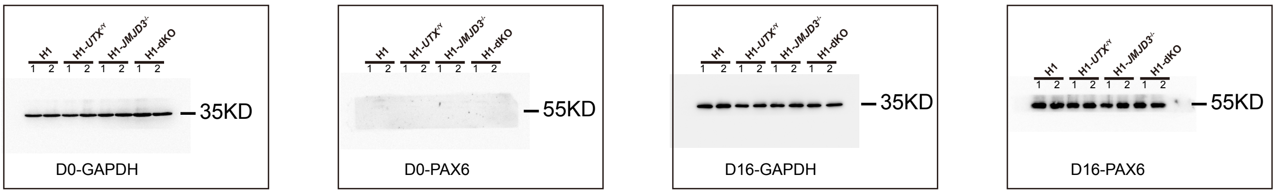

Figure 1g

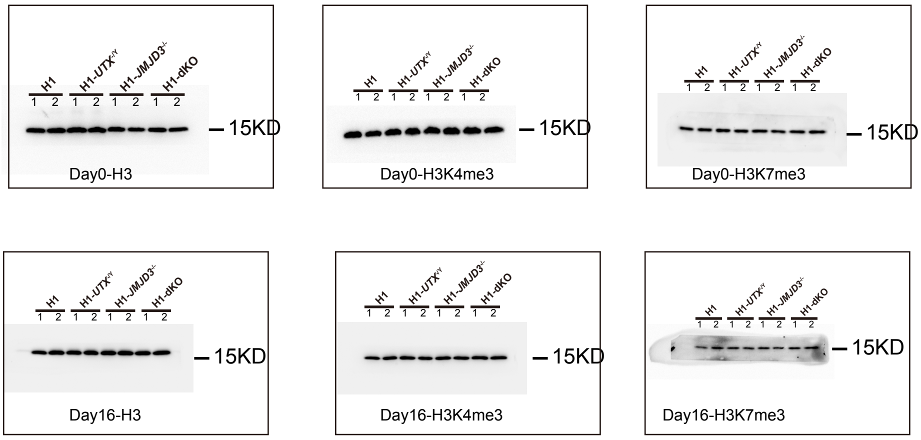

Figure 6a

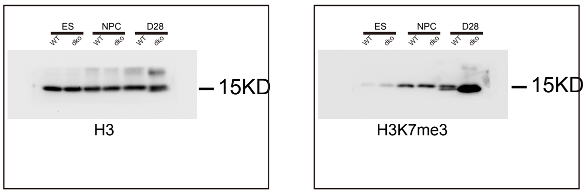

Figure 7a

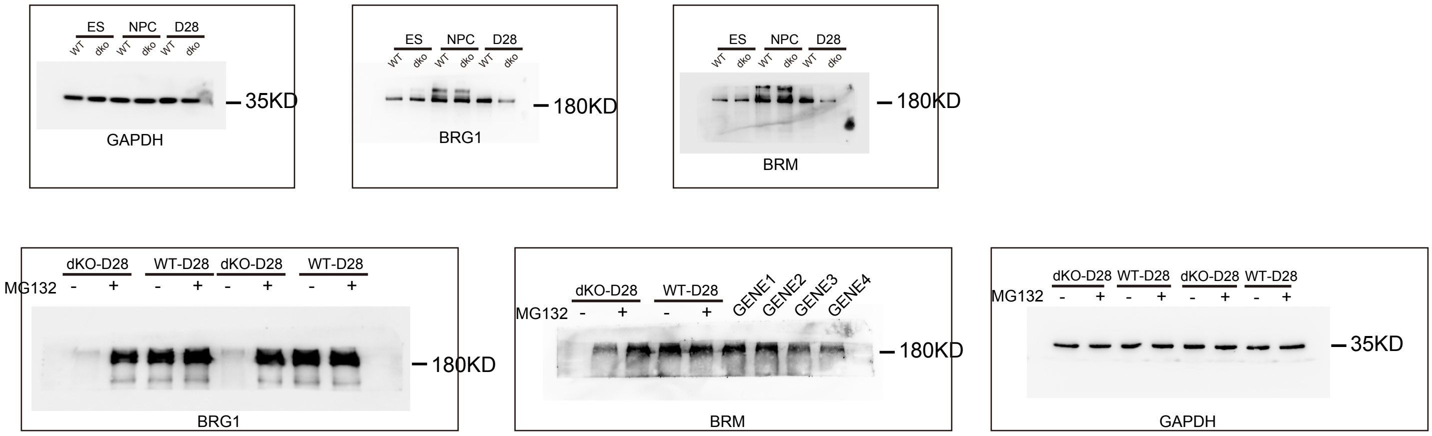

Supplementary Figure 7 The uncropped western blots in this paper

Supplementary Figure 8

Supplementary Figure 6a

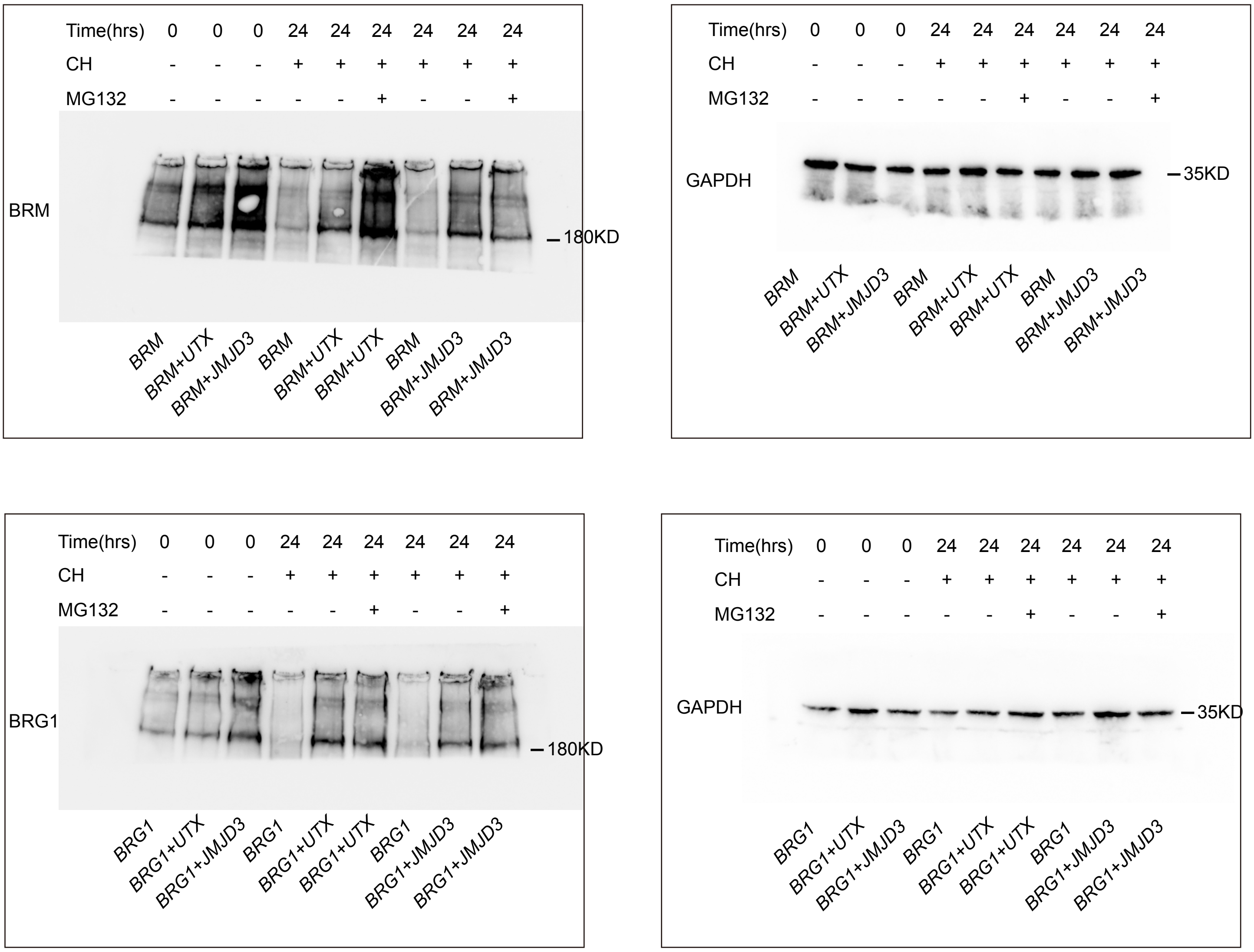

Supplementary Figure 6b

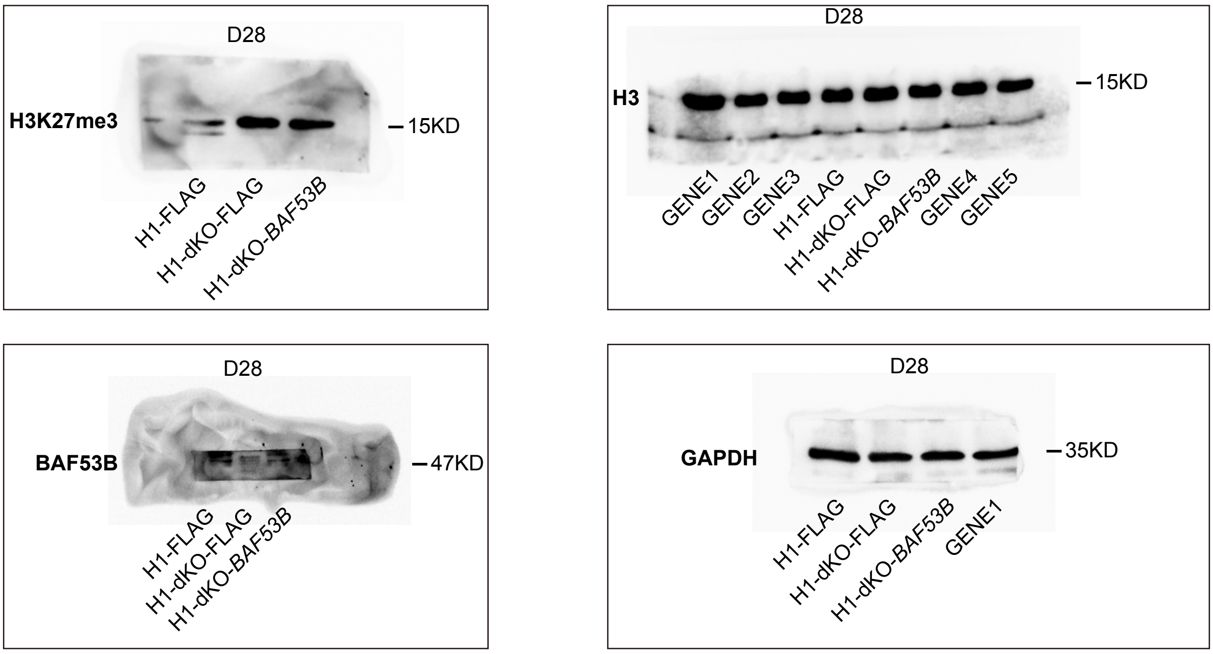

Supplementary Figure 8 The uncropped western blots in this paper

Supplementary Figure 9

Supplementary Figure 1i

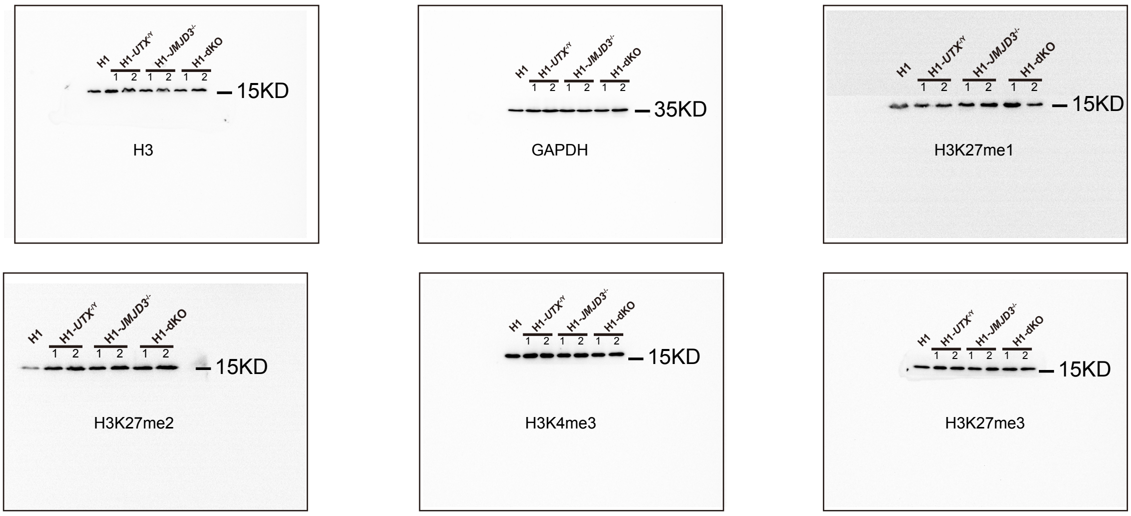

Supplementary Figure 3b

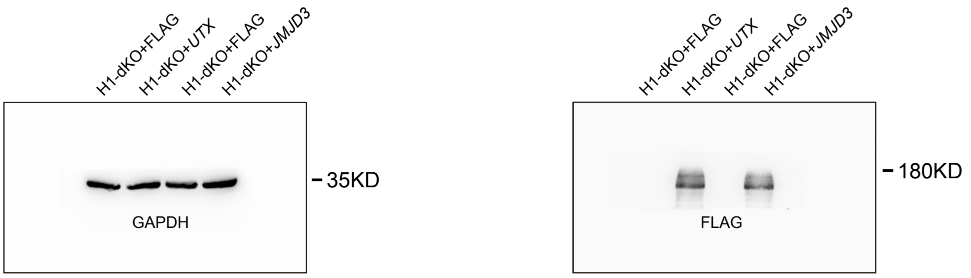

Figure 6d

Figure 6d left upper panel, FLAG WB for 3×FLAG knock-in at C-terminal of *UTX*.

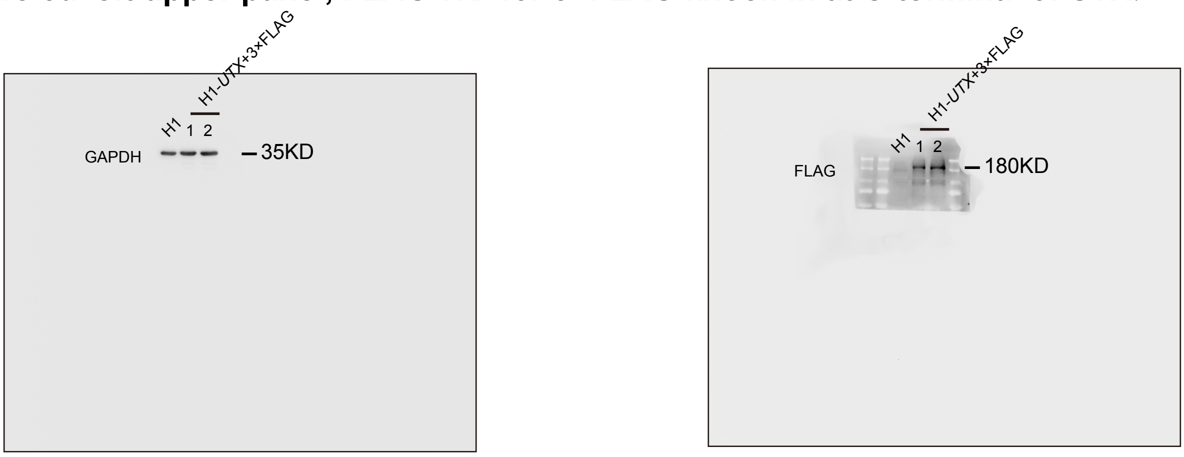

Figure 6d left lower panel, FLAG WB for 3×FLAG knock-in at C-terminal of *JMJD3*.

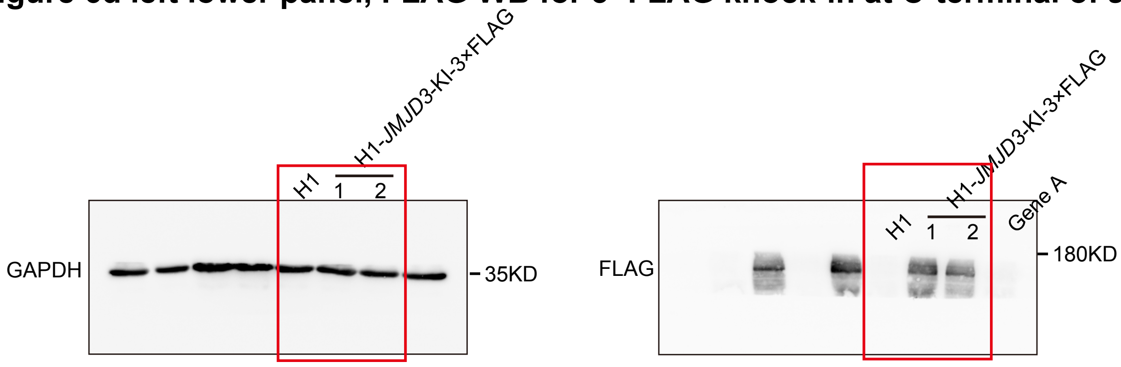

Supplementary Figure 9 The uncropped western blots in this paper

Supplementary Figure 10

Figure 1d

Figure 1d for PAX6 positive cells in Day 16 during neural differentiation from hESCs (%)

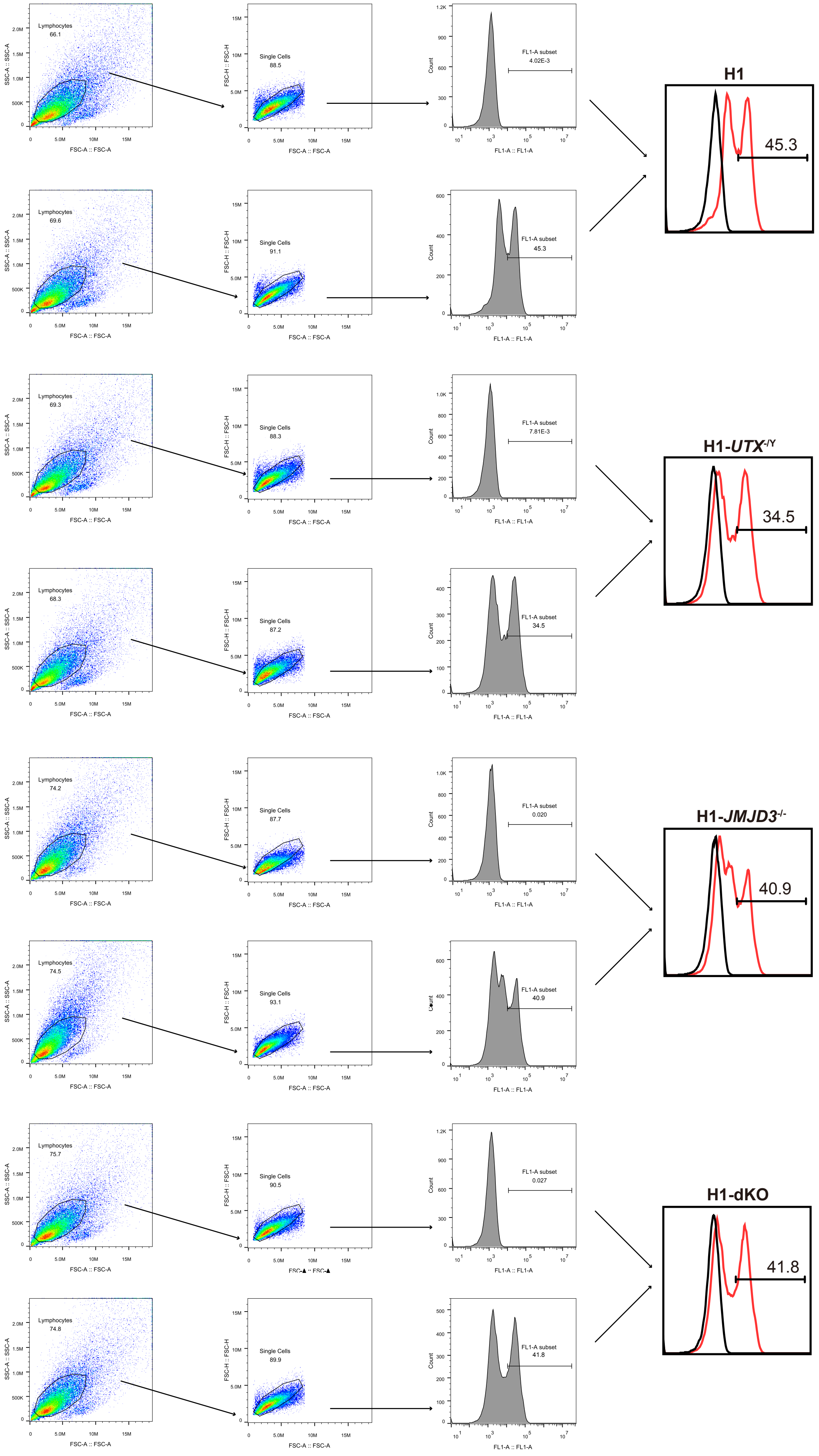

Supplementary Figure 10 The FACS sequential gating strategies in this paper

Supplementary Figure 11

Figure 2e

Figure 2e for apoptosis analysis in indicated NPCs at passage 2 (P2) and passage 4 (P4) (%)

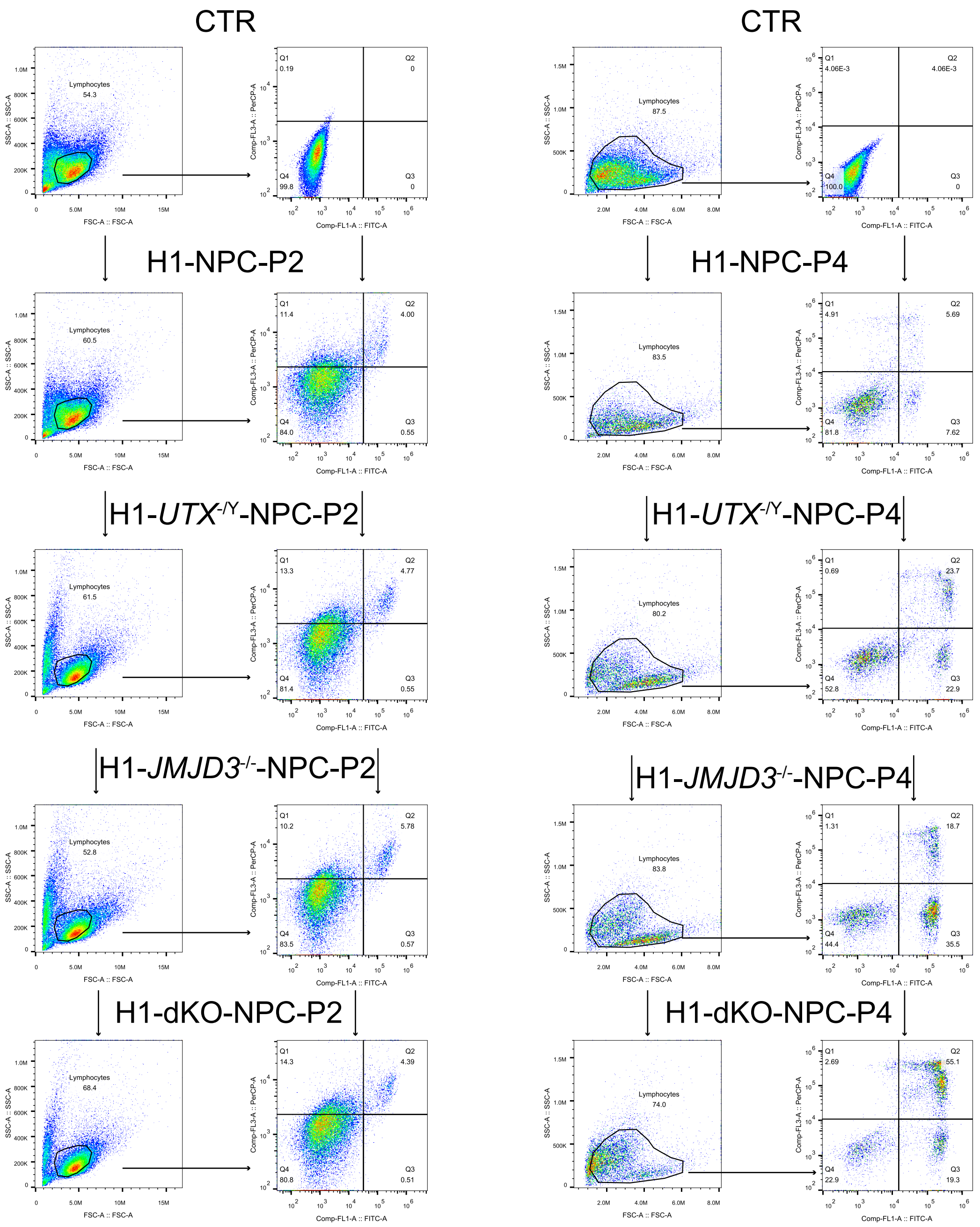

Supplementary Figure 11 The FACS sequential gating strategies in this paper

Supplementary Figure 12

Supplementary Figure 1h

Supplementary Figure 1h for OCT4 and SSEA4 in indicated hESCs (%)

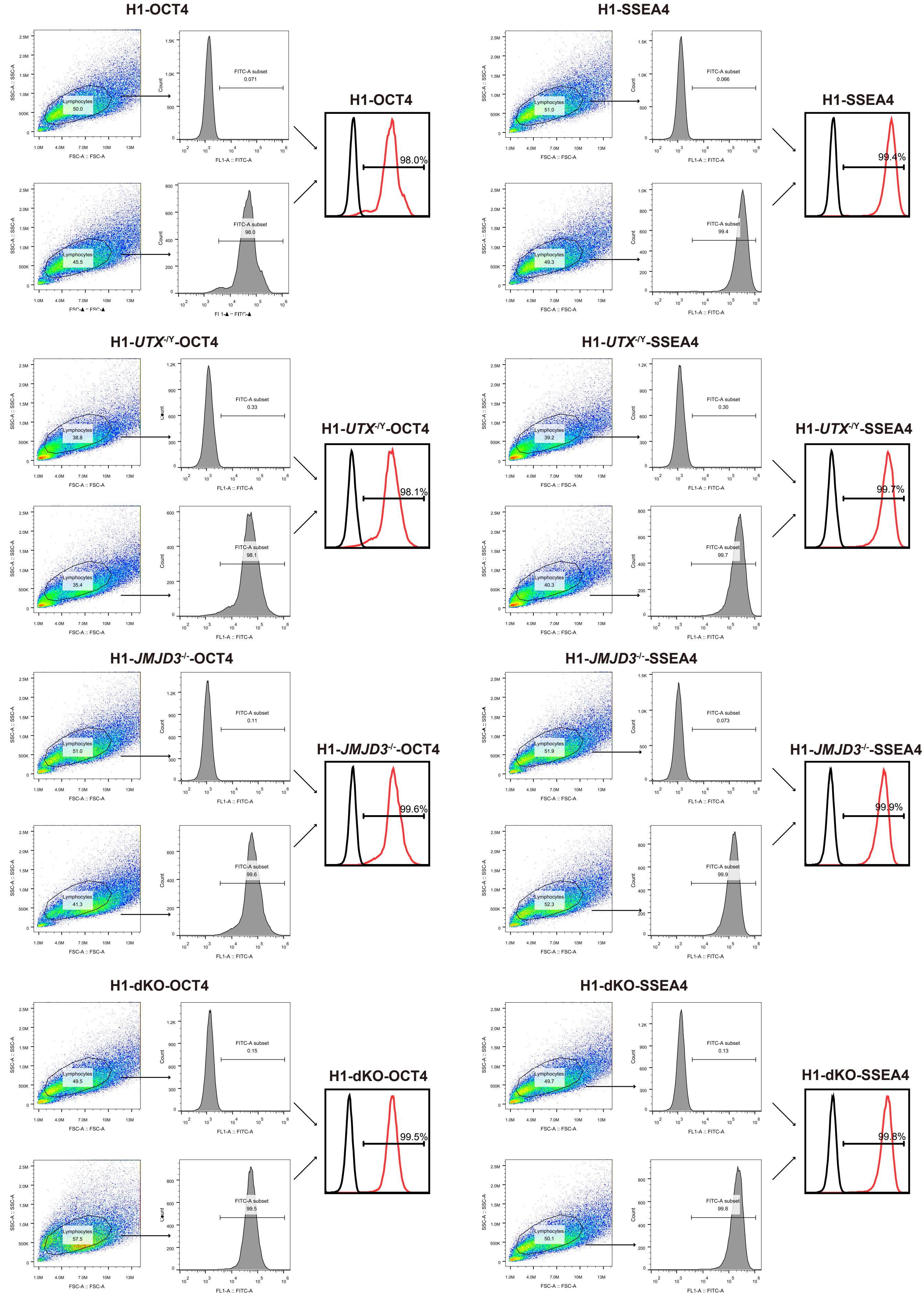

Supplementary Figure 12 The FACS sequential gating strategies in this paper

Supplementary Figure 13

Supplementary Figure 2a

Supplementary Figure 2a for EdU analysis in indicated NPCs at passage 2 (P2) and passage 4 (P4) (%)

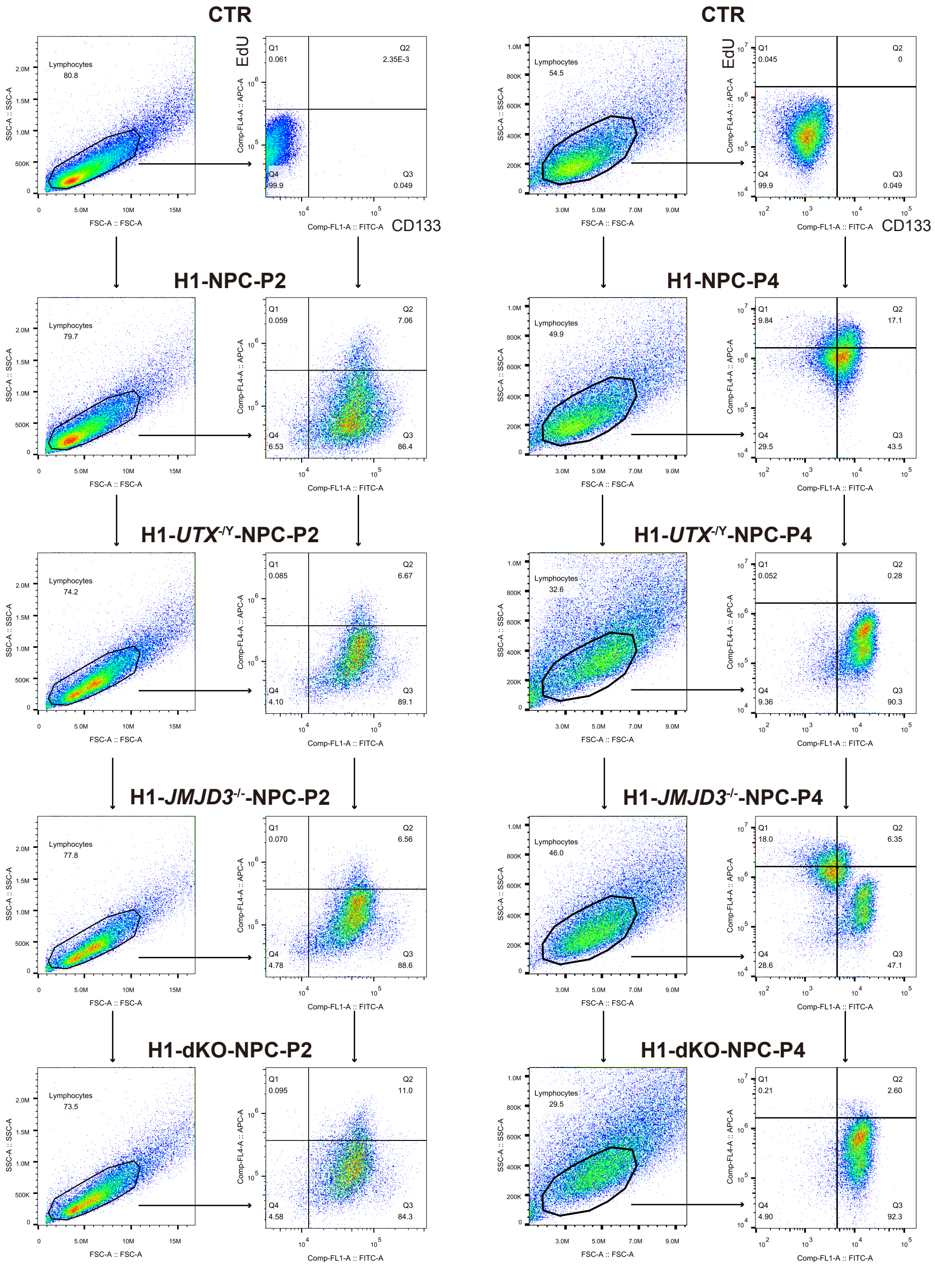

Supplementary Figure 13 The FACS sequential gating strategies in this paper

Supplementary Figure 14

Supplementary Figure 6f

Supplementary Figure 6f for OCT4 and SSEA4 in indicated hESCs

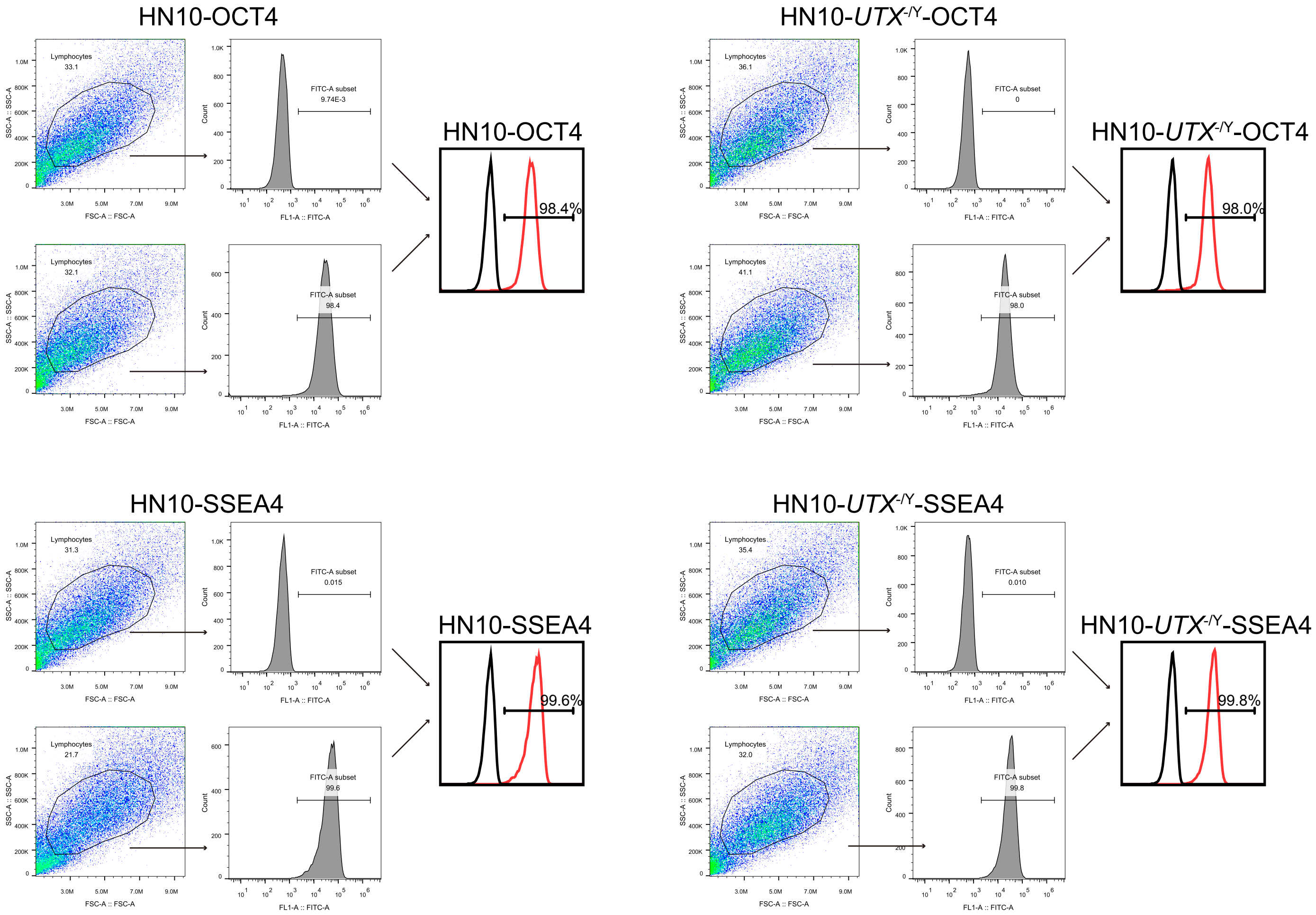

Supplementary Figure 14 The FACS sequential gating strategies in this paper

Supplementary Table 1 List of sgRNAs and primers for gene targeting

Gene	*UTX*		*JMJD3*	
**Knock-out**				
**sgRNA sequence**	TAAACGACAACTTACCAAGC		GCGAACCACTCGCAGTCGCC	
**Primers for validation**	*UTX*-F1	5' CATTCTGCACGCTTCAAAAGCG 3'	*JMJD3*-F1	5' GACCCTCTGAGAAATCCCACACTTG 3'
	UTX-R1	5' CCCTAAAAATAGCCAGTCCCTACTCAG 3'	*JMJD3*-R1	5' ACCGGTGGATGTGGAATGTGTG 3'
	UTX-F2	5' AATGGTTTACCAACTTGCCCAGG 3'	*JMJD3*-F2	5' CTTCATGCGGGTAACATCCACG 3'
	UTX-R2	5' CGGGTGCTCAATAAAGAATTCCTTC 3'	*JMJD3*-R2	5' TTCCAGGCAATGTTGTTGCACC 3'
**Primers for donor DNA construction**	*UTX*-5' arm-F	5' GCTGTACCAATGTACTTGGGGCTG 3'	*JMJD3*-5' arm-F	5' CCGTGAGGAGTCCACTGAGTGATG 3'
	UTX-5' arm-R	5' GGTGCAATTGGTTAGCACACGG 3'	*JMJD3*-5' arm-R	5' ACTACTACAGCTGCACCGTGTTCATGC 3'
	UTX-3' arm-F	5' CGACAACTTACCAAGCTGGATCTCC 3'	*JMJD3*-3' arm-F	5' TAGTAGCCATCAGCGCTTTCTGTGATCG 3'
	UTX-3' arm-R	5' AGTTAGTATGGGGCTGGGCGTG 3'	*JMJD3*-3' arm-R	5' TACCAGGTACGTGTTGCGGCTG 3'
**Knock-in**				
**sgRNA sequence**	GTTGGTTGACACTCCGCACT		CCGGCCTCATCGCGACGTGC	
**Primers for donor DNA construction**	*UTX*-5' arm-F	5' CAGTACAAAATGGAGGACCTGATGC 3'	*JMJD3*-5' arm-F	5' TACTTGACGGGTTCCTGGTGGC 3'
	UTX-5' arm-R	5' TCACTTATCGTCGTCATCCTTGTAATCCTTATCGTCG TCATCCTTGTAATCCTTATCGTCGTCATCCTTGTAATC AGATGAGGCGGATGGTAATGGAG 3'	*JMJD3*-5' arm-R	5' TCACTTATCGTCGTCATCCTTGTAATCCTTATC GTCGTCATCCTTGTAATCCTTATCGTCGTCATC CTTGTAATCTCGCGACGTGCTGGCTGG 3'
	UTX-3' arm-F	5' TATTGTTCCATGGACATTAAATGAGACC 3'	*JMJD3*-3' arm-F	5' ACATGCCTGGGCTGGACCTAG 3'
	UTX-3' arm-R	5' CCCCCAAATCATGTCATGCAAG 3'	*JMJD3*-3' arm-R	5' GTCCTCCCCATCAATCACGGTC 3'

Supplementary Table 2 List of primers for qRT-PCR

Gene	Forward primer	Reward primer
GAPDH	GGAGCGAGATCCCTCCAAAAT	GGCTGTTGTCATACTTCTCATGG
OCT4	CCTCACTTCACTGCACTGTA	CAGGTTTTCTTTCCCTAGCT
SOX2	CCCAGCAGACTTCACATGT	CCTCCCATTTCCCTCGTTTT
NANOG	TGAACCTCAGCTACAAACAG	TGGTGGTAGGAAGAGTAAAG
PAX6	ATGTGTGAGTAAAATTCTGGGCA	GCTTACAACTTCTGGAGTCGCTA
SOX1	AATTTTATTTTCGGCGTTGC	TGGGCTCTGTCTCTTAAATTTGT
NES	GAAGGGCAATCACAACAGGTG	GGGGCCACATCATCTTCCA
UTX	TTGGCCCAGGTGACTGTGAATG	TTCAAGATTGGGCCACCAAGAAC
JMJD3	GCCAGGCCACCAGGAGAATAAC	CTCCCAGTAGTGCTCGTGCACC
CASPASE3	GAAATTGTGGAATTGATGCGTGA	CTACAACGATCCCCTCTGAAAAA
CASPASE6	CACCAACATAACTGAGGTGGATG	AGGAGGAGCCATATTTTCCCA
CASPASE7	AGGGACCGAGCTTGATGATG	CACTGGGATCTTGTATCGAGGA
CASPASE8	AGAGTCTGTGCCCAAATCAAC	GCTGCTTCTCTCTTTGCTGAA
CASPASE9	CTCAGACCAGAGATTCGCAAAC	GCATTTCCCCTCAAACTCTCAA
ROCK1	GGTGGTCGGTTGGGGTATTTT	CGCCCTAACCTCACTTCCC
ROCK2	TCAGAGGTCTACAGATGAAGGC	CCAGGGGCTATTGGCAAAGG
FAS	TCTGGTTCTTACGTCTGTTGC	CTGTGCAGTCCCTAGCTTTCC
P53	GAGGTTGGCTCTGACTGTACC	TCCGTCCCAGTAGATTACCAC
P21	CGATGGAACTTCGACTTTGTCA	GCACAAGGGTACAAGACAGTG
P27	AACGTGCGAGTGTCTAACGG	CCCTCTAGGGGTTTGTGATTCT
NESTIN	CTGGAGCAGGAGAAACAGG	TGGGAGCAAAGATCCAAGAC
FABP7	TTCATGGGCTCAAGCGAT	CATGGCGAAATCCCATCT
VIM	GACGCCATCAACACCGAGTT	CTTTGTCGTTGGTTAGCTGGT
DLX1	TGCCAGAAAGTCTCAACAGCC	CGAGTGTAAACAGTGCATGGA
DLX2	GCCTCAACAACGTCCCTTACT	GGGAGCGTAGGAGGTGTAGG
MAP2	TGAAGCAAAGGCACCTCAC	TATGGGAATCCATTGGCG
TUJ1	GGCCAAGGGTCACTACACG	GCAGTCGCAGTTTTCACACTC
NEUN	CCAAGCGGCTACACGTCTC	CGTCCCATTCAGCTTCTCCC
SLC32A1	ACGTCCGTGTCCAACAAGTC	AAAGTCGAGGTCGTCGCAATG
GBJ6	CAAGAGGACTTCGTCTGCAAC	GTGGTTTCGTGCCTGTAGTAG
GFAP	CTGCGGCTCGATCAACTCA	TCCAGCGACTCAATCTTCCTC
BRG1	CAGATCCGTCACAGGCAAAAT	TCTCGATCCGCTCGTTCTCTT
BRM	AGCGGGAATACAGACTTCAGG	AAGTGCTTTTAGTTCCACGGTT
BAF53A	CTATGCAGTGCAGAGAACTCTTC	CCTCGTAACCTGAGGCAACTT
BAF53B	GCACTACGAGATGCCCAATG	TTGACGTTCGAGGGATCAAAC

Supplementary Table 3 List of antibodies used in this study

Name of Antibody	Company (Cat. No.)	Dilution Factor
Rabbit anti-HIST3H3 (H3)	Abclonal (A2348)	1:1000
Rabbit anti-Histone H3K27me3 (tri-methyl Lys27)	GeneTex (GTX54106)	1:1000
Rabbit anti-TriMethyl-Histone H3-K4 Polyclonal	Abclonal (A2357)	1:1000
HRP-conjugated Monoclonal Mouse Anti-GAPDH	KangChen Bio-tech (KC-5G5)	1:1000
Goat anti Rabbit IgG HRP	KangChen Bio-tech (KC-RB-035)	1:4000
mouse anti-OCT-3/4	Santa Cruz Biotechnology (sc-5279)	1:200
mouse anti-SSEA4	Invitrogen (414000)	1:200
mouse anti-Isotype antibody mouse IgG2b	Invitrogen (MG2800)	1:200
Goat Anti-Mouse IgG H&L (Alexa Fluor® 488)	Abcam (ab150113)	1:500
Rabbit anti-OCT-3/4	Cell Signaling Technology (2750S)	1:200
Rabbit anti-Glial Fibrillary Acidic Protein Antibody, clone GA5	Millipore (MAB360)	1:1000
Goat anti-Rabbit IgG (H+L) Cross-Adsorbed Secondary Antibody, Alexa Fluor 488	Thermo Fisher SCIENTIFIC (A-11008)	1:500
Goat anti-Mouse IgG (H+L) Cross-Adsorbed Secondary Antibody, Alexa Fluor 568	Thermo Fisher SCIENTIFIC (A-11004)	1:500
Donkey anti-Goat IgG (H+L) Cross-Adsorbed Secondary Antibody, Alexa Fluor 568	Thermo Fisher SCIENTIFIC (A-11057)	1:500
Mouse anti-PAX6	BD Biosciences (561664)	1:100
Mouse anti-IgG2a	BD Biosciences (554647)	1:100
Rabbit anti-CD133	proteintech (18470-1-AP)	1:100
Mouse anti-SOX2	R&D system (MAB2018)	1:1000
Rabbit anti-NES	Millipore (ABD69)	1:1000
Mouse anti-Ki67	BD Biosciences (556003)	1:1000
Rabbit anti-PAX6	BioLegend (901301)	1:1000
Mouse anti-MAP2	Millipore (MAB3418)	1:1000
Rabbit anti-GFAP	proteintech (16825-1-AP)	1:500
Rabbit anti-TUBB3	GeneTex (gtx130245)	1:1000
Mouse anti-Annexin V	proteintech (66245-1-IG)	1:500
Rabbit anti-BRM (SMARCA2)	GeneTex (gtx133724)	1:500
mouse anti-BRG1	GeneTex (gtx633391)	1:500
Rabbit anti-BAF53A(ACTL6A)	HUABIO (ET7106-60)	1:500
Rabbit anti-BAF53B	Novus Biologicals (NBP2-15269)	1:1000
Mouse anti-NES	Cell Signaling Technology (33475)	1:500
Rabbit anti-MAP2	Cell Signaling Technology (8707)	1:500
Mouse anti-FLAG	Sigma-Aldrich (F1804)	1:1000
Anti-FLAG M2 magnetic beads ChIP	Sigma-Aldrich (M8823)	25 μL per chromatin immunoprecipitation
Anti-H3K27me3 ChIP	Millipore (17-622)	4 μL per chromatin immunoprecipitation

Supplementary Table 4 List of primer sequences for over-expression and knock-down

Gene	Forward primer	Reverse primer
**Over-expression**		
UTX	ATGAAATCCTGCGGAGTGTCGCT	TCAAGATGAGGCGGATGGTAATGG
JMJD3	ATGCATCGGGCAGTGGACCC	TCATCGCGACGTGCTGGCT
BAF53B	ATGAGCGGGGGCGTCTAC	TCAGGGGCACTTTCGCTCC
BAF53A	ATGAGCGGCGGCGTGTAC	TCAAGGGCATTTTCTTTCTACACAC
BRG1	ATGGAGACCAGCCAGATGAGCGACC	TCAGATTTTCTGCCGCACGCTGG
BRM	ATGCAGAAGATTGAGCAGGAGAGGAAAC	TCAAATTTTCTGCCGGGCACTCT
**Knock-down**		
***BAF53A*-sh1**	GCTTTCCTTGAAATGCACTTACTCGAGTAAGTGCATTTCAAGGAAAGCTTTTT	
***BAF53A*-sh2**	CGGTACTTCAAGTGTCAGATTCTCGAGAATCTGACACTTGAAGTACCGTTTTT	
