# Supplementary material for: JMJD3 and UTX determine fidelity and lineage specification of human neural progenitor cells
Source: Nat Commun. 2020 Jan 20;11:382. doi: 10.1038/s41467-019-14028-x (PMC6971254; doi:10.1038/s41467-019-14028-x)
Supplement: Supplementary file 3 — Reporting Summary [file 41467_2019_14028_MOESM3_ESM.pdf]

nature research

Corresponding author(s): Guangjin Pan

Last updated by author(s): Nov 25, 2019

Reporting Summary

Nature Research wishes to improve the reproducibility of the work that we publish. This form provides structure for consistency and transparency in reporting. For further information on Nature Research policies, see Authors & Referees and the Editorial Policy Checklist.

Statistics

For all statistical analyses, confirm that the following items are present in the figure legend, table legend, main text, or Methods section.

n/a	Confirmed	
☐	☒	The exact sample size (*n*) for each experimental group/condition, given as a discrete number and unit of measurement
☐	☒	A statement on whether measurements were taken from distinct samples or whether the same sample was measured repeatedly
☐	☒	The statistical test(s) used AND whether they are one- or two-sided<br>*Only common tests should be described solely by name; describe more complex techniques in the Methods section.*
☐	☒	A description of all covariates tested
☒	☐	A description of any assumptions or corrections, such as tests of normality and adjustment for multiple comparisons
☐	☒	A full description of the statistical parameters including central tendency (e.g. means) or other basic estimates (e.g. regression coefficient) AND variation (e.g. standard deviation) or associated estimates of uncertainty (e.g. confidence intervals)
☒	☐	For null hypothesis testing, the test statistic (e.g. *F*, *t*, *r*) with confidence intervals, effect sizes, degrees of freedom and *P* value noted<br>*Give P values as exact values whenever suitable.*
☒	☐	For Bayesian analysis, information on the choice of priors and Markov chain Monte Carlo settings
☒	☐	For hierarchical and complex designs, identification of the appropriate level for tests and full reporting of outcomes
☐	☒	Estimates of effect sizes (e.g. Cohen's *d*, Pearson's *r*), indicating how they were calculated

Our web collection on statistics for biologists contains articles on many of the points above.

Software and code

Policy information about availability of computer code

Data collection	In general, results were presented as mean ± SD calculated using Microsoft Excel and GraphPad Prism at least three biological repeats. Significance level between samples was determined using unpaired two-tailed Student’s t-tests. P value <0.05 was considered statistically significant in the figures. No samples were excluded for any analysis.<br>For FACS, cells were collected on a BD Accuri C6 Plus.<br>For RNA-seq, ChIP-seq, ATAC-seq, our data collection processes were described in methods and materials section of this manuscript.
Data analysis	We used Microsoft Excel and GraphPad Prism to analyze these data.<br>For FACS, these data were analyzed using flowjo.<br>For RNA-seq, ChIP-seq, ATAC-seq, our data analysis processes were described in methods and materials section of this manuscript.

For manuscripts utilizing custom algorithms or software that are central to the research but not yet described in published literature, software must be made available to editors/reviewers. We strongly encourage code deposition in a community repository (e.g. GitHub). See the Nature Research guidelines for submitting code & software for further information.

Data

Policy information about availability of data

All manuscripts must include a data availability statement. This statement should provide the following information, where applicable:

- Accession codes, unique identifiers, or web links for publicly available datasets
- A list of figures that have associated raw data
- A description of any restrictions on data availability

The RNA-Seq, ATAC-seq, ChIP-seq data have been deposited in the Gene Expression Omnibus database under the accession code GSE118999 and GSE133209. A reporting summary for this Article is available as a Supplementary Information file. PCR and qRT-PCR data have also been deposited in figshare (https://doi.org/10.6084/m9.figshare.8035574). The source data underlying Figures 1c, 2d, 2f, 3e and 7a-c and Supplementary Figures 1e,1f, 2c,6c and 6e are provided as a Source Data file. The authors declare that all data supporting the findings of this study are available within the article and its supplementary information files or

from the corresponding author (Dr. Guangjin Pan, pan_guangjin@gibh.ac.cn) upon reasonable request.

Field-specific reporting

Please select the one below that is the best fit for your research. If you are not sure, read the appropriate sections before making your selection.

☒ Life sciences ☐ Behavioural & social sciences ☐ Ecological, evolutionary & environmental sciences

For a reference copy of the document with all sections, see nature.com/documents/nr-reporting-summary-flat.pdf

Life sciences study design

All studies must disclose on these points even when the disclosure is negative.

Sample size	In this study, human embryonic stem cells, neural progenitor cells, differentiated neural progenitor cells were used. No statistical methods were used to determine sample size. We chose the sample size based on literatures in the field.
Data exclusions	No exclusions.
Replication	Phenotypes observed are robust and were reliably reproduced at least three biological repeats. RNA-seq data and ATAC-seq data were analyzed with two repeats. ChIP-seq data were performed one time.
Randomization	In this study, we analyzed cell morphology, qRT-PCR data, Western blot data, cell proliferation, EdU assay and apoptosis assay, RNA-seq data, ChIP-seq data, and ATAC-seq data with cell populations. We analyzed immuno-staining data with three random selected pictures.
Blinding	No blinding was used in this study.

Reporting for specific materials, systems and methods

We require information from authors about some types of materials, experimental systems and methods used in many studies. Here, indicate whether each material, system or method listed is relevant to your study. If you are not sure if a list item applies to your research, read the appropriate section before selecting a response.

Materials & experimental systems

n/a	Involved in the study	
☐	☒	Antibodies
☐	☒	Eukaryotic cell lines
☒	☐	Palaeontology
☒	☐	Animals and other organisms
☒	☐	Human research participants
☒	☐	Clinical data

Methods

n/a	Involved in the study	
☐	☒	ChIP-seq
☐	☒	Flow cytometry
☒	☐	MRI-based neuroimaging

Antibodies

Antibodies used

The detail information of antibodies used in this study was listed in Supplementary Table 3. Please see Supplementary Table 3.

Validation

Rabbit anti-HIST3H3 (H3), Abclonal, Cat. A2348; 1:1,000; western blot validation and peer-reviewed citations at https://abclonal.com.cn/catalog/A2348.
Rabbit anti-Histone H3K27me3 (tri-methyl Lys27), Genetex, Cat. GTX54106; 1:1,000; western blot validation and peer-reviewed citations at http://www.genetex.com/Histone-H3K27me3-tri-methyl-Lys27-antibody-GTX54106.html.
Rabbit anti-TriMethyl-Histone H3-K4 Polyclonal, Abclonal, Cat. A2357; 1:1,000; western blot validation and peer-reviewed citations at https://abclonal.com.cn/catalog/A2357.
HRP-conjugated Monoclonal Mouse Anti-GAPDH, KangChen Bio-tech, Cat. KC-5G5; 1:1,000; western blot validation and peer-reviewed citations at http://www.aksomics.com/index.php?c=article &id=1160.
Goat anti Rabbit IgG HRP, KangChen Bio-tech, Cat. KC-RB-035; 1:4,000; western blot validation and peer-reviewed citations at http://www.aksomics.com/products/secondary-antibody.html.
mouse anti-OCT-3/4, Santa Cruz Biotechnology, Cat. sc-5279; 1:200; FACS validation and peer-reviewed citations at https://www.scbt.com/scbt/product/oct-3-4-antibody-c-10?requestFrom=search#thumbcarousel.
mouse anti-SSEA4, Invitrogen, Cat. 414000; 1:200; FACS validation and peer-reviewed citations at https://www.thermofisher.com/antibody/product/SSEA4-Antibody-clone-MC813-70-Monoclonal/41-4000.
mouse anti-Isotype antibody mouse IgG2b, Invitrogen, Cat. MG2800; 1:200; FACS validation. This antibody is no longer produced.
Goat Anti-Mouse IgG H&L (Alexa Fluor® 488), Abcam, Cat. ab150113; 1:500; FACS validation and peer-reviewed citations at https://www.abcam.cn/goat-mouse-igg-hl-alexa-fluor-488-ab150113.html.
Rabbit anti-OCT-3/4 Cell Signaling Technology, Cat. 2750S; 1:200; immune-staining validation and peer- reviewed citations at https://www.cst-c.com.cn/products/primary-antibodies/oct-4-antibody/2750?_=1534692337046&Ntt=2750S&tahead=true.
Rabbit anti-Glial Fibrillary Acidic Protein Antibody, clone GA5, Millipore, Cat. MAB360; 1:1000; immune-staining validation and

peer- reviewed citations at https://www.sigmaaldrich.com/catalog/product/mm/mab360?lang=zh&region=CN.
Goat anti-Rabbit IgG (H+L) Cross-Adsorbed Secondary Antibody, Alexa Fluor 488, Thermo Fisher SCIENTIFIC, Cat. A-11008; 1:500, immune-staining validation and peer- reviewed citations at https://www.thermofisher.com/antibody/product/Goat-anti-Rabbit-IgG-H-L-Cross-Adsorbed-Secondary-Antibody-Polyclonal/A-11008.
Goat anti-Mouse IgG (H+L) Cross-Adsorbed Secondary Antibody, Alexa Fluor 568, Thermo Fisher SCIENTIFIC, Cat. A-11004; 1:500; immune-staining validation and peer- reviewed citations at https://www.thermofisher.com/antibody/product/Goat-anti-Mouse-IgG-H-L-Cross-Adsorbed-Secondary-Antibody-Polyclonal/A-11004.
Donkey anti-Goat IgG (H+L) Cross-Adsorbed Secondary Antibody, Alexa Fluor 568, Thermo Fisher SCIENTIFIC, Cat. A-11057; 1:500; immune-staining validation and peer- reviewed citations at https://www.thermofisher.com/antibody/product/Donkey-anti-Goat-IgG-H-L-Cross-Adsorbed-Secondary-Antibody-Polyclonal/A-11057.
Mouse anti-PAX6, BD Biosciences, Cat. 561664; 1:100; immune-staining validation and peer-reviewed citations at https://www.bdbiosciences.com/cn/applications/research/intracellular-flow/intracellular-antibodies-and-isotype-controls/anti-human-antibodies/alexa-fluor-488-mouse-anti-human-pax-6-o18-1330/p/561664.
Mouse anti-IgG2a, BD Biosciences, Cat. 554647; 1:100; immune-staining validation and peer-reviewed citations at https://www.bdbiosciences.com/cn/reagents/research/antibodies-buffers/immunology-reagents/anti-human-antibodies/cell-surface-antigens/fitc-mouse-igg2a-isotype-control-g155-178/p/554647.
Rabbit anti-CD133, proteintech, Cat. 18470-1-AP; 1:100; immune-staining validation and peer-reviewed citations at https://www.ptglab.com/products/PROM1-Antibody-18470-1-AP.htm.
Mouse anti-SOX2, R&D system, Cat. MAB2018; 1:1000; immune-staining validation and peer-reviewed citations at https://www.rndsystems.com/cn/products/human-mouse-rat-sox2-antibody-245610_mab2018.
Rabbit anti-NES, Millipore, Cat. ABD69; 1:1000; immune-staining validation and peer- reviewed citations at https://www.sigmaaldrich.com/catalog/product/mm/abd69?lang=zh&region=CN.
Mouse anti-Ki67, BD Biosciences, Cat. 556003, 1:1000; immune-staining validation and peer-reviewed citations at http://www.bdbiosciences.com/cn/applications/research/intracellular-flow/intracellular-antibodies-and-isotype-controls/anti-rat-antibodies/purified-mouse-anti-ki-67-b56/p/556003.
Rabbit anti-PAX6, BioLegend, Cat.901301; 1:1000; western blot validation and peer-reviewed citations at https://www.biolegend.com/en-us/products/purified-anti-pax-6-antibody-11511.
Mouse anti-MAP2, Millipore, Cat. MAB3418; 1:1000; immune-staining validation and peer- reviewed citations at https://www.sigmaaldrich.com/catalog/product/mm/mab3418?lang=zh&region=CN.
Rabbit anti-GFAP, proteintech, Cat. 16825-1-AP; 1:500; immune-staining validation and peer-reviewed citations at https://www.ptglab.com/products/GFAP-Antibody-16825-1-AP.htm.
Rabbit anti-TUBB3, GeneTex, Cat. gtx130245; 1:1000; immune-staining validation and peer-reviewed citations at http://www.genetex.com/beta-Tubulin-3-Tuj1-antibody-GTX130245.html.
Mouse anti-Annexin V, proteintech, Cat. 66245-1-IG; 1:500; immune-staining validation and peer- reviewed citations at https://www.ptglab.com/products/Annexin-V-Antibody-66245-1-Ig.htm.
Mouse anti-NES, Cell Signaling Technology, Cat. 33475; 1:500; immune-staining validation and peer-reviewed citations at https://www.cst-c.com.cn/products/primary-antibodies/nestin-10c2-mouse-mab/33475?_=1534693100419&Ntt=33475&tahead=true.
Rabbit anti-MAP2, Cell Signaling Technology, Cat. 8707; 1:500; immune-staining validation and peer- reviewed citations at https://www.cst-c.com.cn/products/primary-antibodies/map2-d5g1-xp-rabbit-mab/8707?_=1534692344705&Ntt=8707&tahead=true.
Mouse anti-FLAG, Sigma-Aldrich, Cat. F1804, 1:1000; western blot validation and peer-reviewed citations at https://www.sigmaaldrich.com/catalog/product/sigma/f1804?lang=zh&region=CN.
Rabbit anti-BRM (SMARCA2), GeneTex, Cat. gtx133724, 1:500; western blot validation and peer-reviewed citations at https://www.genetex.com/Product/Detail/SMARCA2-antibody/GTX133724.
Rabbit anti-BRG1, GeneTex, Cat. gtx633391, 1:500; western blot validation and peer-reviewed citations at https://www.genetex.com/Product/Detail/BRG1-antibody-GT2712/GTX633391.
Rabbit anti-BAF53A, HUABIO, Cat. ET7106-60, 1:500/1:100; western blot and immune-staining validation and peer-reviewed citations at http://www.huabio.cn/product1_det.php?idd=27609.
Rabbit anti-BAF53B, Novus Biologicals, Cat. NBP2-15269, 1:1000/1:200; western blot and immune-staining validation and peer-reviewed citations at https://www.novusbio.com/products/actl6b-antibody_nbp2-15269.
H3K27me3 antibody for ChIP-seq assay, Millipore, Cat. 17-622; The detail information of this antibody is available as https://www.merckmillipore.com/CN/zh/product/ChIPAb+-Trimethyl-Histone-H3-Lys27-ChIP-Validated-Antibody-and-Primer-Set,MM_NF-17-622.
Anti-FLAG M2 magnetic beads for ChIP-seq assay, Sigma-Aldrich, Cat. M8823; The detail information of this antibody is available as https://www.sigmaaldrich.com/catalog/product/sigma/m8823?lang=zh&region=CN.

Eukaryotic cell lines

Policy information about cell lines

Cell line source(s)	H1 hESCs were purchased from Wi Cell. HN10 hESCs were kindly provided by our collaborative colleagues in Hainan provincial key Laboratory for human reproductive medicine and genetic research.
Authentication	These cell lines have been authenticated by karyotyping.
Mycoplasma contamination	We have tested these cell lines for mycoplasma contamination. We found that these cell lines are not contamination with mycoplasma.
Commonly misidentified lines (See ICLAC register)	N/A

ChIP-seq

Data deposition

☒ Confirm that both raw and final processed data have been deposited in a public database such as GEO.

☒ Confirm that you have deposited or provided access to graph files (e.g. BED files) for the called peaks.

Data access links<br>*May remain private before publication.*	GSE118999, GSE133209
Files in database submission	H1_NPC_H3K27me3-R1.fastq.gz<br>H1_D28_H3K27me3-R1.fastq.gz<br>dKO_NPC_H3K27me3-R1.fastq.gz<br>dKO_D28_H3K27me3-R1.fastq.gz<br>H1-NPC-INPUT-R1.fastq.gz<br>H1-D28-INPUT-R1.fastq.gz<br>dKO-NPC-INPUT-R1.fastq.gz<br>dKO-D28-INPUT-R1.fastq.gz<br>H1-NPC-UTX-FLAG-R1.fastq.gz<br>H1-NPC-INPUT-R1.fastq.gz<br>H1-NPC-JMJD3-FLAG-R1.fastq.gz<br>H1-NPC-INPUT-JMJD3-R1.fastq.gz
Genome browser session<br>(e.g. UCSC)	N/A

Methodology

Replicates	ChIP-seq were performed on NPC and differentiated NPC at day 28 of dKO and WT cells with one time.
Sequencing depth	On average 20 million 75-bp single-end reads per library were mapped and 70% of the reads were uniquely mapped to the reference genome.<br>H1-D28-INPUT, 24559847 reads, 18306128 (74.54%) aligned exactly 1 time.<br>H1-D28-H3K27me3, 31315431 reads, 19730558 (63.01%) aligned exactly 1 time.<br>H1-NPC-INPUT, 21163022 reads, 15289499 (72.25%) aligned exactly 1 time.<br>H1-NPC-H3K27me3, 28470826 reads, 18434246 (64.75%) aligned exactly 1 time.<br>dKO-D28-INPUT, 26277864 reads, 19134366 (72.82%) aligned exactly 1 time.<br>dKO-D28--H3K27me3, 42763934 reads, 28994128 (67.80%) aligned exactly 1 time.<br>dKO-NPC-INPUT, 28828724 reads, 21323126 (73.96%) aligned exactly 1 time.<br>dKO-NPC-H3K27me3, 35376160 reads, 21005965 (59.38%) aligned exactly 1 time.<br>H1-NPC-UTX-FLAG, 130778798 reads, 98807624 (75.55%) aligned exactly 1 time.<br>H1-NPC-INPUT,76815763 reads, 58001856 (75.51%) aligned exactly 1 time.<br>H1-NPC-JMJD3-FLAG: 171107658 reads; 126382140 (73.86%) aligned exactly 1 time.<br>H1-NPC-INPUT-JMJD3: 47282607 reads; 36284292 (76.74%) aligned exactly 1 time.
Antibodies	In this study, we used H3K27me3 antibody to do ChIP-seq assay. This antibody was purchased from Millipore. Catalog number is 17-622. Immunogen is KLH-conjugated, synthetic 2X-branched peptide containing the sequence AR(me3K)SAP. Lot number is 2907374. The detail information of this antibody is available as https://www.merckmillipore.com/CN/zh/product/ChIPAb+-Trimethyl-Histone-H3-Lys27-ChIP-Validated-Antibody-and-Primer-Set,MM_NF-17-622.<br>We used anti-FLAG M2 magnetic beads to do UTX and JMJD3 ChIP-seq assay. This antibody was purchased from Sigma-Aldrich. Catalog number is M8823. The detail information of this antibody is available as https://www.sigmaaldrich.com/catalog/product/sigma/m8823?lang=zh&region=CN.
Peak calling parameters	For H3K27me3 ChIP, the regions of H3K27me3 enrichment (peaks) were called using the SICER software package, with the input genomic DNA as a background control (parameters: W = 200; G = 600; FDR 0.01 cutoff applied). Binding profiles and heatmaps were generated using ngsplot and deeptools.<br>For UTX-FLAG and JMJD3-FLAG ChIP, we performed peak calling using the MACS2(2.1.0) callpeak module with parameters "-p 0.1 --nomodel --extsize 150 -B --SPMR --keep-dup all --call-summits" on transcription factor ChIP-seq data, and then only peaks with qvalue less than 0.05 were keeped. Tracks of signal were computed using MACS2 bdgcmp module with parameter "-m ppois".
Data quality	For ChIP-seq data, we confirmed that quality control results were good following the ENCODE guideline and reads were converted to bigwig files. We observed clear peaks with inspection on Integrated Genomics Viewer (Broad Institute) and there was high consistency. Read counts per million were calculated.
Software	Bowtie2, SICER software package, ngsplot, deeptools.

Flow Cytometry

Plots

Confirm that:

☒ The axis labels state the marker and fluorochrome used (e.g. CD4-FITC).

☒ The axis scales are clearly visible. Include numbers along axes only for bottom left plot of group (a 'group' is an analysis of identical markers).

☒ All plots are contour plots with outliers or pseudocolor plots.

☒ A numerical value for number of cells or percentage (with statistics) is provided.

Methodology

Sample preparation	Accutase (sigma) was used to digested sample cells to single cells, and then these single cells were fixed in fixation buffer (BD Bioscеinces) at room temperature for about 20 minutes. After fixation, these cells were washed in PBS, and then were permeabilizated in perm/wash buffer (BD Bioscеinces) for 10-15 minutes at 4 °C. After washed with PBS, these cells and corresponding primary antibodies were incubated together at 37 °C for 30 minutes. Meanwhile, other cell samples were incubated with corresponding isotype control antibodies at 37 °C for 30 minutes. After incubation, cells were washed with PBS. And then these samples were incubated with secondary antibodies at 37 °C for 30 minutes. After washed twice, these cells were resuspended with PBS, and used for FACS analysis.<br>The detail information of FACS analysis for EdU assay, cell cycle and apoptosis assay were described in methods, Please see methods.
Instrument	These samples were analyzed with Accuri C6 (BD Biosciences).
Software	These data were collected with Accuri C6 (BD Biosciences) and analyzed with Flow Jo.
Cell population abundance	50,000 cells.
Gating strategy	Cell debris were excluded by FSC-A/SSC-A plot and singlets were gated by SSC-A/SSC-W plot. The gates for positive cells were determined by non staining cells.

☒ Tick this box to confirm that a figure exemplifying the gating strategy is provided in the Supplementary Information.
